# Tislelizumab induced dual organs dysfunction in a patient with advanced esophageal squamous cell carcinoma: a case report

**DOI:** 10.3389/fonc.2024.1347896

**Published:** 2024-03-14

**Authors:** Bo Yang, Wei Gou, Naiying Lan, Qing Shao, Weifeng Hu, Cheng Xue, Nanmei Liu

**Affiliations:** ^1^ Internal Medicine III (Nephrology), Naval Medical Center of PLA, Naval Medical University, Shanghai, China; ^2^ Department of Nephrology and Endocrinology, Shanghai 411 Hospital, Shanghai University, Shanghai, China; ^3^ Division of Nephrology, Changzheng Hospital, Naval Medical University, Shanghai, China

**Keywords:** tislelizumab, acute kidney injury, acute liver injury, immune checkpoint inhibitor, esophageal squamous cell carcinoma

## Abstract

**Background:**

Tislelizumab, a humanized IgG4 anti-PD-1 monoclonal antibody has been approved in China and Europe. According to the published clinical research, tislelizumab shows satisfactory safety profile. No severe hepatotoxicity or acute kidney injury were reported.

**Case presentation:**

We presented a case study of a 74-year-old man who developed acute kidney injury (grade 3) and acute liver injury (grade 4) after being administered tislelizumab for the treatment of esophageal squamous cell carcinoma. We reviewed the patient’s history, physical examination, and laboratory findings and provided comprehensive differentials of the possible causes of the toxicities. Immune Checkpoint Inhibitors (ICI) hepatotoxicity and nephrotoxicity were confirmed clinically. We also discussed the management of toxicities associated with ICIs and the need for a multidisciplinary approach to care.

**Conclusions:**

The case highlights the importance of close monitoring and prompt management of toxicities associated with ICIs and the need for further research to better understand the risk factors for these toxicities and to identify effective treatments for them.

## Introduction

Esophageal squamous cell carcinoma (ESCC) is a type of cancer derived from the epithelial cells of the esophagus. It is one of the most common types of cancer globally with a high mortality rate. According to the Global Cancer Observatory, there were approximately 500,000 new cases of ESCC reported globally in 2020. The incidence of ESCC has also been shown to vary across different regions, with the highest rates found in eastern to central Asia, along the rift valley in east Africa (1).

The current treatment options of ESCC includes surgery, chemotherapy, radiotherapy, and immunotherapy. These treatments are used depending on the stage of the cancer, the patient’s general health, and the preference of the patient and their doctor. For the advanced and metastatic ESCC, the goals of therapy are to palliate symptoms and improve survival and immunotherapy plays an active role. For advanced or metastatic ESCC patients, the use of pembrolizumab and nivolumab are related to a significant survival benefit. Even higher level of evidence suggested the use of PD-1 inhibitors for patients with certain MSI profile and PD-L1 testing results (2, 3).

Tislelizumab, a humanized IgG4 anti-PD-1 monoclonal antibody produced by BeiGene, Inc. has been approved in China and Europe for ESCC but has not been approved by US FDA. According to the published clinical research, tislelizumab shows beneficial overall survival and satisfactory safety profile in the ESCC population (4–7). The most common grade 3 or 4 treatment-related adverse events were decreased neutrophil/WBC count and anemia. The product information of tislelizumab in Europe revealed that immune-related nephritis and renal dysfunction (greater than grade 2) happened in 0.4% of patient using tislelizumab monotherapy, the median time from first dose to renal adverse events was 1.2 months. 50% of the renal injury recovered in 3 days to 16.2 months (8). No severe hepatotoxicity or acute kidney injury (AKI) was reported in the clinical trials (4–7). Herein, we report a case who has been administrated tislelizumab and developed severe dual organ dysfunction (kidney and liver). Written informed consent for this study was obtained from the patient himself.

## Case presentation

On Aug. 8^th^, 2023, a 74-year-old Asian man presented to the local oncology center due to dysphagia and underwent esophageal endoscopy. Biopsies taken during the scope revealed ESCC in the mid-esophagus. The patient has no comorbidities and no long-term treatments. The following evaluation including Positron emission tomography–computed tomography (PET-CT) suggested the stage of cTxN2M0. The oncology center recommended neoadjuvant therapy, which consisted of tislelizumab 200 mg on day 1 + albumin-bound paclitaxel 156 mg on days 1, 8, and 15 + carboplatin 400 mg on day 1. The patient completed the first cycle of therapy (day 1: Aug. 23^rd^, 2023). After the rescue of thrombocytopenia, it was scheduled to start the second cycle on Sept. 20^th^. However, on arrival at the clinic on Sept. 20^th^, the patient was found to have slightly elevated liver enzymes (Alanine transaminase (ALT) 89 U/L (reference range 7-40 U/L), Aspartate Aminotransferase (AST) 68 U/L (reference range 13-35 U/L)), with total bilirubin at 136.11 µmol/L (reference range 3.4-17.1 µmol/L), Direct bilirubin (DBIL) at 90.6 µmol/L (reference range 0-5 µmol/L), Serum creatinine (SCr) at 304 µmol/L (reference range 41-81 µmol/L), and Blood urea nitrogen (BUN) at 19.2 mmol/L (reference range 3.1-8.9) (**Table 1**). The patient also had jaundiced skin and sclera. The patient reported no abdominal pain, bloating, diarrhea, vomiting, oliguria, skin rashes, joint pain, or photosensitivity. The patient also reported that in the past 3-4 days, the stool had been light-colored. Then the patient was referred to the nephrologist in Naval Medical Center of PLA. The latest biochemistry results (Sept. 9^th^) were also provided. Physical examination was unremarkable other than jaundice. The patient was diagnosed with AKI stage 3 and acute liver injury and was hospitalized. The following examinations including abdominal ultrasound, renal vascular ultrasound, urine routine test, blood lipid profile, Magnetic resonance cholangiopancreatography (MRCP), and viral hepatitis screening were ordered to figure out the causes of renal and liver injury. The results suggested generally normal blood routine tests and urine routine tests (except for positive urinary bilirubin). No evidence of glomerular diseases, pre-/post-renal diseases was detected according to the clinical features, normal urine routine test, urinary system and renal vascular ultrasound examination. Acute tubulointerstitial disease was suspected. The most possible reason would be nephrotoxic exposure. In terms of liver injury, liver enzyme tests suggested cholestatic pattern abnormality (R<2) [(ALT/Upper limit of normal ALT)/(Alkaline phosphatase/Upper limit of normal Alkaline phosphatase)] (9). No evidence of extrahepatic cholestasis was found through ultrasound and MRCP. Viral hepatitis screening results were all negative. Drug-related intrahepatic cholestasis was suspected.

**Table 1:** Lab test results provided on Sept. 20^th^.

	Hb (g/L)	PLT (*10^9/L)	WBC (*10^9/L)	ALT (U/L)	AST (U/L)	ALP (U/L)	GGT (U/L)	TB (umol/L)	DBIL (umol/L)	SCr (umol/L)	BUN (mmol/L)	alb (g/L)
	115-150	125-350	3.69-9.16	7-40	13-35	50-135	7-40	3.4-17.1	0-5	41-81	3.1-8.9	40-55
**2023-08-16**	134	101	4.9	20	31	92	32	10.5	2.1	83	6	40
**2023-08-25**	119	97	5.7	/	/	/	/	/	/	/	/	/
**2023-09-01**	106	98	3.2	28	30	/	/	13.1	3.2	98	7.1	37
**2023-09-04**	109	101	1.62	/	/	/	/	/	/	/	/	/
**2023-09-09**	117	72	3.3	25	28	/	/	10.4	2.4	92	9.1	40
**2023-09-11**	107	62	2.7	/	/	/	/	/	/	/	/	/
**2023-09-14**	113	70	7.7	/	/	/	/	/	/	/	/	/
**2023-09-15**	106	69	7.7	/	/	/	/	/	/	/	/	/
**2023-09-18**	102	78	6.9	/	/	/	/	/	/	/	/	/
**2023-09-20**	102	120	6.8	89	68	/	/	136.1	90.6	304	19.2	31

Hb, hemoglobin; PLT, platelet count; WBC, white blood cell count; ALT, alanine transaminase, AST, aspartate aminotransferase, ALP, alkaline phosphatase; GGT, gamma-glutamyl Transferase; TB, Total bilirubin; DB, Direct bilirubin; SCr, serum creatinine; BUN, Blood Urea Nitrogen; alb, albumin. * to multiply; / no data.

**Figure 1:**
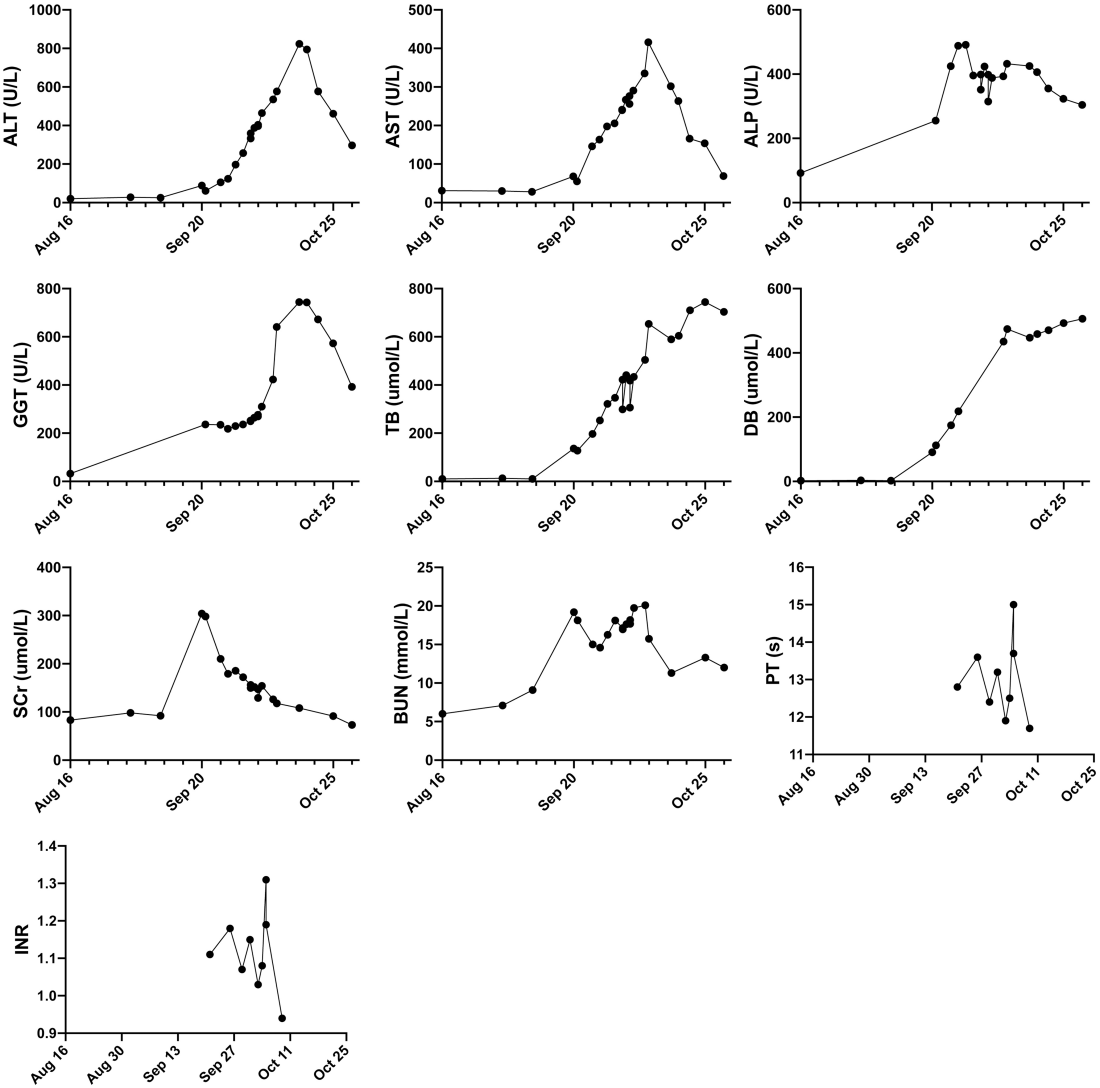
Follow-up of the major lab test results over time. ALT, alanine transaminase (reference range 7-40 U/L), AST, aspartate aminotransferase (reference range 13-35 U/L), ALP: alkaline phosphatase (reference range 50-135 U/L); GGT, gamma-glutamyl Transferase (reference range 7-40 U/L); TB, Total bilirubin (reference range 3.4-17.1 umol/L); DB, Direct bilirubin (reference range 0-5 umol/L); SCr, serum creatinine (reference range 41-81 umol/L); BUN, Blood Urea Nitrogen (reference range 3.1-8.9 mmol/L); PT, prothrombin time (reference range 9-13s).

Then we reviewed the history of immunotherapy and chemotherapy. Four weeks before the onset of kidney and liver injury, the patient was administrated tislelizumab, carboplatin, and albumin-bound paclitaxel. Two more doses of albumin-bound paclitaxel were administrated in the following two weeks. No other medications or herb supplements were taken. Nephrotoxicity of platinum agents is well established while AKI from platinum typically manifests with a rise of SCr after three to seven days of exposure (10–12). Carboplatin is significantly less nephrotoxic than cisplatin (13, 14). Paclitaxel undergoes minimal renal excretion. It is usually safe for the kidneys. Limited data on animals showed acute (24-48h) renal toxicity (15). It is unlikely for the patient to experience acute renal dysfunction after weeks of platinum/Paclitaxel administration. It should be noted that tislelizumab falls under the category of immune checkpoint inhibitors (ICIs), which allows us to draw some inferences regarding its potential side effects. After excluding other causes of abnormal liver/renal tests, ICI hepatotoxicity and nephrotoxicity were confirmed clinically. According to the guideline on ICI adverse events management (16), we gave methylprednisolone 48 mg qd from Sept. 27^th^. Basic metabolic panels were monitored closely. After 7-day treatment, renal function was restored somewhat, while there was no improvement in liver enzymes and bilirubin (**Figure 1**). Dual plasma molecular adsorption system was applied four times from Oct. 3^rd^. Mycophenolate mofetil 0.5g bid was also started to treat the refractory hepatotoxicity. On Oct. 7^th^, the patient was referred to a hepatologist with expertise in ICI hepatitis. Phone call follow-ups were conducted regularly after that. In the following days, basic metabolic panels were also showed in **Figure 1**. Mycophenolate mofetil was discontinued at Oct. 23 while methylprednisolone was still keep going. At Nov 1^st^, the patient developed signs of hospital-acquired pneumonia. Invasive gillosis was confirmed based on the chest imaging and airway specimen. The patient did not respond to the antifungals and died at Nov. 5^th^.

## Discussion

ICIs have been a paradigm shift in the treatment of many types of cancer. These drugs work by blocking the receptors on T cells that keep the immune system from attacking the cancer cells (17). This has resulted in significant improvements in the prognosis of many cancer patients, particularly those with metastatic or advanced disease (18). Several ICIs have been approved by the FDA for the treatment of multiple types of cancer. However, despite these promising results, ICIs can also cause a range of side effects, including autoimmune disorders and gastrointestinal side effects. These toxicities can be managed with the use of corticosteroids, immune modulators, and supportive care (19).

Toxicities associated with tislelizumab are known to occur, due to the nature of ICIs. However, the incidence and severity of these toxicities may vary between individuals. In the case of the patient we reported, the patient experienced severe dual-organ toxicities, including AKI (grade 3) and acute liver toxicity (grade 4), which have not been previously reported in association with tislelizumab. This case highlighted the importance of close monitoring and prompt management of toxicities associated with ICIs, particularly when using a new drug or in combination with other therapies. In clinical practice, patients often use a variety of medications and herb supplements, which can result in complex medication interactions. Those confounding factors lead to delayed diagnosis of ICI adverse events and potentially even requiring a biopsy to accurately determine the source of the adverse reaction. It also underscored the need for further research to better understand the mechanisms, risk factors for these toxicities and to identify effective treatments for them.

The renal outcome of the toxicities due to ICIs is not well studied. While the management of toxicities associated with ICIs has been well-established, the management of renal complications is not fully evidence-based. In other words, while there are guidelines and protocols for managing these toxicities, the evidence for these approaches is lacking. Besides, kidney histopathology is heterogeneous due to ICI toxicity (20). Glomerular capillaries, mesangial matrix/cells, podocytes and tubule could be possible target of activated immune cells (21–24). More clinical studies are needed in this field to improve our understanding of the renal outcome and to provide evidence-based treatments for these complications. It is important to note that the management of renal complications due to ICIs can be challenging, and a collaborative approach between nephrologists and oncologists is essential for achieving the best possible outcomes for patients. In addition, close monitoring and prompt management of toxicities associated with ICIs will remain critical for ensuring their safe and effective use in the treatment of cancer.

The risk factors associated with experiencing renal/liver injury due to ICI use are not yet entirely clear. While there is some evidence suggesting that certain factors, such as type/dose of ICIs and underlying malignancy, may increase the risk (25, 26), more research is needed to fully understand the risk factors. This is particularly important given the growing use of ICIs in the treatment of cancer patients. As more information becomes available about the risks associated with ICI use, it is important for healthcare providers to carefully consider these risks when prescribing ICIs to patients. Healthcare providers should also be familiar with the signs and symptoms of renal/liver injury and should monitor patients closely for these toxicities. The management of toxicities associated with ICI use requires a multidisciplinary approach.

In summary, more research is needed to fully understand the risk factors for renal/liver injury associated with ICI use, and healthcare providers should carefully weigh the benefits and risks of ICI use in cancer treatment, while also being prepared to manage any toxicities that may arise.

## Data availability statement

The original contributions presented in the study are included in the article/supplementary material. Further inquiries can be directed to the corresponding authors.

## Ethics statement

The studies involving humans were approved by Institutional Review Committee of the Naval Medical Center. The studies were conducted in accordance with the local legislation and institutional requirements. Written informed consent for participation in this study was provided by the participants’ legal guardians/next of kin. Written informed consent was obtained from the individual(s) for the publication of any potentially identifiable images or data included in this article.

## Author contributions

BY: Funding acquisition, Writing – original draft, Writing – review & editing, Data curation. WG: Writing – review & editing, Data curation, Investigation. NYL: Data curation, Investigation, Writing – review & editing. QS: Data curation, Investigation, Writing – review & editing. WH: Data curation, Investigation, Writing – review & editing. CX: Writing – review & editing, Writing – original draft. NML: Conceptualization, Writing – review & editing.

## References

[1] Abnet CC Arnold M Wei W-Q. Epidemiology of esophageal squamous cell carcinoma. Gastroenterology. (2018) 154:360–73. doi: 10.1053/j.gastro.2017.08.023 PMC5836473 28823862

[2] Liu Y. Perioperative immunotherapy for esophageal squamous cell carcinoma: Now and future. World J Gastroenterol. (2023) 29:5020–37. doi: 10.3748/wjg.v29.i34.5020 PMC10518742 37753366

[3] Ajani JA D’Amico TA Bentrem DJ Cooke D Corvera C Das P. Esophageal and esophagogastric junction cancers, version 2.2023, NCCN clinical practice guidelines in oncology. J Natl Compr Cancer Netw. (2023) 21:393–422. doi: 10.6004/jnccn.2023.0019 37015332

[4] Shen L Kato K Kim S-B Ajani JA Zhao K He Z. Tislelizumab versus chemotherapy as second-line treatment for advanced or metastatic esophageal squamous cell carcinoma (RATIONALE-302): A randomized phase III study. J Clin Oncol. (2022) 40:3065–76. doi: 10.1200/JCO.21.01926 PMC9462531 35442766

[5] Yoon H Kato K Raymond E Hubner R Shu Y Pan Y. LBA-1 RATIONALE-306: Randomized, global, placebo-controlled, double-blind phase 3 study of tislelizumab plus chemotherapy versus chemotherapy as first-line treatment for advanced or metastatic esophageal squamous cell carcinoma (ESCC). Ann Oncol. (2022) 33:S375. doi: 10.1016/j.annonc.2022.04.439 37080222

[6] Jin P sheng GY Fu Z feng YW Meng X. Neoadjuvant tislelizumab combined with chemoradiotherapy for resectable locally advanced esophageal squamous cell carcinoma (ESCC): Single arm phase II study. J Clin Oncol. (2023) 41:4068–8. doi: 10.1200/JCO.2023.41.16_suppl.4068

[7] Xu J Kato K Raymond E Hubner RA Shu Y Pan Y. Tislelizumab plus chemotherapy versus placebo plus chemotherapy as first-line treatment for advanced or metastatic oesophageal squamous cell carcinoma (RATIONALE-306): a global, randomised, placebo-controlled, phase 3 study. Lancet Oncol. (2023) 24:483–95. doi: 10.1016/S1470-2045(23)00108-0 37080222

[8] Tevimbra product information . Available online at: https://www.ema.europa.eu/en/documents/product-information/tevimbra-epar-product-information_en.pdf (Accessed February 28, 2024).

[9] Kalas MA Chavez L Leon M Taweesedt PT Surani S. Abnormal liver enzymes: A review for clinicians. World J Hepatol. (2021) 13:1688–98. doi: 10.4254/wjh.v13.i11.1688 PMC8637680 34904038

[10] McSweeney KR Gadanec LK Qaradakhi T Ali BA Zulli A Apostolopoulos V. Mechanisms of cisplatin-induced acute kidney injury: pathological mechanisms, pharmacological interventions, and genetic mitigations. Cancers (Basel). (2021) 13:1572. doi: 10.3390/cancers13071572 33805488 PMC8036620

[11] McMahon KR Rassekh SR Schultz KR Blydt-Hansen T Cuvelier GDE Mammen C. Epidemiologic characteristics of acute kidney injury during cisplatin infusions in children treated for cancer. JAMA Netw Open. (2020) 3:e203639. doi: 10.1001/jamanetworkopen.2020.3639 32383745 PMC7210480

[12] Asai A Katsuno T Yamaguchi M Iwagaitsu S Nobata H Kinashi H. Carboplatin-related acute interstitial nephritis in a patient with pancreatic neuroendocrine tumor. CEN Case Rep. (2020) 9:114–21. doi: 10.1007/s13730-019-00437-w PMC7148392 31834568

[13] Ries F Klastersky J. Nephrotoxicity induced by cancer chemotherapy with special emphasis on cisplatin toxicity. Am J Kidney Dis. (1986) 8:368–79. doi: 10.1016/s0272-6386(86)80112-3 3538860

[14] McDonald BR Kirmani S Vasquez M Mehta RL. Acute renal failure associated with the use of intraperitoneal carboplatin: a report of two cases and review of the literature. Am J Med. (1991) 90:386–91. doi: 10.1016/0002-9343(91)80022-E 2003521

[15] Rabah SO. Acute Taxol nephrotoxicity: Histological and ultrastructural studies of mice kidney parenchyma. Saudi J Biol Sci. (2010) 17:105–14. doi: 10.1016/j.sjbs.2010.02.003 PMC3730725 23961065

[16] Schneider BJ Naidoo J Santomasso BD Lacchetti C Adkins S Anadkat M. Management of immune-related adverse events in patients treated with immune checkpoint inhibitor therapy: ASCO guideline update. J Clin Oncol. (2021) 39:4073–126. doi: 10.1200/JCO.21.01440 34724392

[17] Li B Chan HL Chen P. Immune checkpoint inhibitors: basics and challenges. Curr Med Chem. (2019) 26:3009–25. doi: 10.2174/0929867324666170804143706 28782469

[18] Shiravand Y Khodadadi F Kashani SMA Hosseini-Fard SR Hosseini S Sadeghirad H. Immune checkpoint inhibitors in cancer therapy. Curr Oncol. (2022) 29:3044–60. doi: 10.3390/curroncol29050247 PMC9139602 35621637

[19] Durrechou Q Domblides C Sionneau B Lefort F Quivy A Ravaud A. Management of immune checkpoint inhibitor toxicities. Cancer Manag Res. (2020) 12:9139–58. doi: 10.2147/CMAR.S218756 PMC7533913 33061607

[20] Tian R Liang J Li R Zhou X. Acute kidney injury induced by immune checkpoint inhibitors. Kidney Dis. (2022) 8:190–201. doi: 10.1159/000520798 PMC9149491 35702709

[21] Fadel F El Karoui K Knebelmann B. Anti-CTLA4 antibody–induced lupus nephritis. N Engl J Med. (2009) 361:211–2. doi: 10.1056/NEJMc0904283 19587352

[22] Charmetant X Teuma C Lake J Dijoud F Frochot V Deeb A. A new expression of immune checkpoint inhibitors’ renal toxicity: when distal tubular acidosis precedes creatinine elevation. Clin Kidney J. (2020) 13:42–5. doi: 10.1093/ckj/sfz051 PMC7025331 32082551

[23] Cortazar FB Marrone KA Troxell ML Ralto KM Hoenig MP Brahmer JR. Clinicopathological features of acute kidney injury associated with immune checkpoint inhibitors. Kidney Int. (2016) 90:638–47. doi: 10.1016/j.kint.2016.04.008 PMC4983464 27282937

[24] Wang R Das T Takou A. IgA nephropathy after pembrolizumab therapy for mesothelioma. BMJ Case Rep. (2020) 13:e237008. doi: 10.1136/bcr-2020-237008 PMC7705558 33257374

[25] Remash D Prince DS McKenzie C Strasser SI Kao S Liu K. Immune checkpoint inhibitor-related hepatotoxicity: A review. World J Gastroenterol. (2021) 27:5376–91. doi: 10.3748/wjg.v27.i32.5376 PMC8409159 34539139

[26] Gérard AO Barbosa S Parassol N Andreani M Merino D Cremoni M. Risk factors associated with immune checkpoint inhibitor–induced acute kidney injury compared with other immune-related adverse events: a case–control study. Clin Kidney J. (2022) 15:1881–7. doi: 10.1093/ckj/sfac109 PMC9494514 36158153

